# Poor power quality is a major barrier to providing optimal care in special neonatal care units (SNCU) in Central India

**DOI:** 10.12688/gatesopenres.13479.1

**Published:** 2022-04-27

**Authors:** Lisa J Messersmith, Alyana Ladha, Cherryl Kolhe, Archana Patel, James S Summers, Sowmya R Rao, Prabir Das, Marym Mohammady, Emily Conant, Nithya Ramanathan, Patricia L Hibberd

**Affiliations:** 1 Global Health, Boston University School of Public Health, Boston, MA, 02118, USA; 2 Lata Medical Research Foundation, Nagpur, India; 3 Datta Meghe Institute of Medical Sciences, Wardha, India; 4 Just Normlicht, Inc, Philadelphia, PA, 19047, USA; 5 NexLeaf Analytics, Los Angeles, CA, 90064, USA

**Keywords:** neonatal health care, special neonatal care units, neonatal intensive care units, India, hospital staff, barriers to care

## Abstract

**Background: **Approximately 25% of all neonatal deaths worldwide occur in India. The Indian Government has established Special Neonatal Care Units (SNCUs) in district and sub-district level hospitals to reduce neonatal mortality, but mortality rates have stagnated. Reasons include lack of personnel and training and sub-optimal quality of care. The role of medical equipment is critical for the care of babies, but its role in improving neonatal outcomes has not been well studied.

**Methods: **In a qualitative study, we conducted seven focus group discussions with SNCU nurses and pediatric residents and thirty-five key informant interviews and with pediatricians, residents, nurses, annual equipment maintenance contractors, equipment manufacturers, and Ministry of Health personnel in Maharashtra between December 2019 and November 2020. The goal of the study was to understand challenges to SNCU care. In this paper, we focus on current gaps and future needs for SNCU equipment, quality of the power supply, and use of SNCU equipment.

**Results: **Respondents described a range of issues but highlighted poor power quality as an important cause of equipment malfunction. Other concerns were lack of timely repair that resulted in needed equipment being unavailable for neonatal care. Participants recommended procuring uninterrupted power supply (UPS) to protect equipment, improving quality/durability of equipment to withstand constant use, ensuring regular proactive maintenance for SNCU equipment, and conducting local power audits to discern and address the causes of power fluctuations.

**Conclusions: **Poor power quality and its negative impact on equipment function are major unaddressed concerns of those responsible for the care and safety of babies in SNCUs in Central India. Further research on the power supply and protection of neonatal equipment is needed to determine a cost-effective way to improve access to supportive care in SNCUs and desired improvements in neonatal mortality rates.

## Introduction

In India, 600,000 neonates die every year, mostly from preventable causes ^1^. The proportion of neonatal deaths in children under age five years increased from 48% to 59% between 2000 and 2016 ^1^. The Indian Ministry of Health and Family Welfare aims to increase the proportion of births in health facilities and, beginning in 2003, established Special Neonatal Care Units (SNCUs) in district and sub-district level hospitals to reduce neonatal mortality for small and sick babies. SNCUs provide Level II care to neonates: 24-hour care by trained neonatologists, staff nurses, and support staff for infants born after 32 weeks’ gestation and weigh more than 1500g at birth ^2^. These infants are typically physiologically immature and moderately ill but expected to recover quickly; they also do not need subspecialty services on an urgent basis. Medical equipment in the SNCU includes radiant warmers, phototherapy units, devices that deliver continuous positive airway pressure (CPAP), and incubators ^3^.

Previous research has focused on shortages of personnel in SNCUs ^4^, the need for improvements in training of SNCU staff ^5^, and improved quality of care, particularly compliance with evidence-based guidelines. A neglected opportunity for improvement is the role of medical equipment to optimize staff support and efficiency, a strategy that frequently benefits patient outcomes ^6, 7^.

Medical equipment is critical for the supportive care of babies in the SNCUs. Suboptimal functioning of equipment or inability to use the equipment has led to adverse outcomes in neonates ^8^. The Indian Government recommends the following measures be adopted for general maintenance and optimal functioning of equipment: provide power back up, conduct regular SNCU power and equipment audits, train staff in equipment usage and preventive equipment maintenance, maintain technical support systems of equipment technicians and engineers, and outsource maintenance services when appropriate ^8^. UNICEF’s toolkit for establishing SNCUs provides specifications related to electrical supply, including ensuring that each piece of SNCU equipment has 24-hour uninterrupted stabilized power supply, a backup power supply, and a generator ^9^. To date, there is little evidence as to whether these specifications are being implemented in SNCUs.

To address this gap in knowledge, we conducted a qualitative study in Maharashtra between December 2019 and November 2020 to better understand the barriers and facilitators to providing optimal care for neonates in the SNCUs and the gaps and future needs for SNCU equipment. This paper focuses on the quality of the power supply, its effect on the functioning of the equipment, and the specific equipment-related needs of front-line health care workers.

## Methods

### Design

To explore power quality and equipment-related barriers to care for neonates in the SNCUs, we conducted Focus Group Discussions (FGD) and Key Informant Interviews (KII) with care providers, hospital and government administrators, equipment manufacturers and maintenance contractors.

### Data collection

***Focus Group Discussions (FGDs) and Key Informant Interviews (KIIs).*** The research team purposively selected nine hospital facilities including district general hospitals, sub-district hospitals, trust hospitals, and medical colleges with SNCUs, Neonatal Intensive Care Units (NICUs), and Specialized Newborn Units (SNBUs). Participants in the focus group discussion (FGD) and key informant interviews (KII) included pediatricians, pediatric medical residents, medical officers, and nurses who consented to be interviewed and were over the age of 18. KII participants also included SNCU/NICU equipment distributors, annual maintenance contractors, equipment designers, and equipment manufacturers who worked in the study districts and were over the age of 18. A total of 66 individuals (35 key informants and 31 FGD participants) participated in the study (please see Table 1 below for more detail). The research team conducted seven FGDs (two with pediatric residents (three and four participants) and five with SNCU staff nurses (four to six participants in each). One in-charge nurse at one facility refused to participate in a KII and did not give a reason. While two FGDs were planned for another facility, these were cancelled due to a staff shortage caused by COVID. FGDs and KIIs explored perspectives on equipment function and use, gaps and needs for medical equipment for different types of newborn units, and the impact of power quality on optimal function of the equipment

**Table 1.** Participants in Key Informant Interviews and Focus Groups by facility type.

Qualitative data collection by facility type							
		General hospital * (n=3)	Sub- district hospitals (n=2)	Trust hospitals * (n=1)	Medical colleges * (n=3)	Other	Total
**Key Informant Interviews**	Hospital administrator	2	-	1	2	-	**5**
	Pediatrician	4	-	1	3	-	**8**
	Medical officer	3	2	-	1	-	**6**
	In-charge sister	2	-	2	3	-	**7**
	Staff nurse	-	2	-	-	-	**2**
	Maintenance contractor	-	-	-	-	2	**2**
	Equipment manufacturer	-	-	-	-	3	**3**
	Ministry of Health personnel	-	-	-	-	2	**2**
**Focus Group Discussions**	Residents	-	-	-	7	-	**7**
	Staff nurses	10	-	5	9	-	**24**
**Total participants**		**21**	**4**	**9**	**25**	**7**	**66**

*Denotes presence of SNCU

The research team developed, pilot-tested and revised FGD and KII guides. FGDs and KIIs were conducted on site at each facility by trained study staff (one interviewer, co-author Cherryl Kohle, MSc, MPH, LMRF Research Associate, and a notetaker) with expertise in qualitative research methods and analysis. Initial meetings between the research staff and the hospital staff were initiated to build rapport and schedule interviews at convenient times. At each site, the newborn care unit director or his/her designate determined which staff were eligible for interviews based on the eligibility criteria above. The research team arranged meetings with eligible participants where the study goals and procedures were explained. Participants were introduced to the study interviewer who is an experienced qualitative researcher in the healthcare field. Participants were informed that there would be no adverse consequences of not participating. Those interested underwent informed consent procedures and, if they agreed to participate, they were asked to sign the written study consent form. FGDs and KIIs were conducted in private areas at each facility and audio taped. Participants were compensated for their time with a payment of approximately USD 10. The interviewer and notetaker met after each interview to write a summary of the interview process. Each participant participated once, and no repeat interviews or FGDs were conducted. All transcripts were de-identified for analysis.

This study was approved by the Boston University Institutional Review Board (H-38641), the Lata Medical Research Foundation Institutional Review Board (RPC #31), and the Indian Council of Medical Research (2019–7899/F1).

### Data analysis

The study team used a Grounded Theory ^10^ approach to understand SNCU staff perspectives on the use and functionality of the newborn care unit equipment in India. Interviews were conducted in English or Marathi and audio-recorded for transcription. All interviews in Marathi were transcribed and translated verbatim from Marathi to English. Each transcript was uploaded into NVivo V.12 (RRID:SCR_014802) (An open-access alternative is R Stats (R Project for Statistical Computing, RRID:SCR_001905)) and coded using line-by-line coding followed by focused coding to identify major themes emerging from the data, a common approach using Grounded Theory. Three coders (one PhD medical anthropologist and two Master’s level public health research assistants) read through the initial 10 transcripts to develop a codebook with parent, child, and grandchild codes and to ensure consistency across coders. The team updated and revised the codebook after reviewing all transcripts and then used the final codebook to code all the transcripts. The team used the constant comparative method to establish analytical distinction and make comparisons throughout the analysis. The team identified themes, patterns, and relationships between themes, and as themes emerged from the data, compared them across transcripts. We used the socio-ecological model as a guiding framework to identify themes, patterns, and relationships between themes regarding equipment and power-related barriers and facilitators to providing optimal care for neonates in the SNCUs at the individual, interpersonal, facility, and systems levels (underlying data ^11^ and extended data ^12^ can be found on Zenodo). Due to study procedures and the Covid-19 lockdown, transcripts were not shared with participants for corrections and findings were not presented to participants for feedback.

### Study sample

Table 1 provides the number of participants by facility type, participant type, and method (either FGD or KII). A total of 66 individuals participated in the study, including 35 key informants and 31 participants in seven FGDs. 

## Results

The participants in our study discussed several equipment-related barriers and facilitators to providing optimal care for babies in the newborn care units. 

### Major themes: equipment-related barriers to providing optimal care for babies in the newborn care units

The following two major themes emerged from the KIIs with equipment manufacturers, AMC contractors, and Ministry of Health personnel as well as KIIs and FGDs with clinical staff and administrators working in the nine study facilities:

- Power sags, surges, and spikes lead to equipment compromise and malfunction.
- Poor quality equipment and constant use lead to frequent breakdowns and need for repair.

***Power sags, surges, and spikes lead to equipment compromise and malfunction.*** Disturbances in power quality such as sags (rapid short-term voltage decrease), surges (rapid short-term voltage increase), spikes or impulses (extremely high increase in voltage within a duration measured in microseconds), and outages (complete power loss for any period of time) can lead to the breakdown of equipment over time ( https://www.gradianhealth.org/). While unit staff do not perceive the quality of the electrical supply to be a problem, AMC contractors and equipment manufacturers are concerned that sags, surges, and spikes in the power, especially frequent outside of metropolitan areas, can damage equipment. These events are particularly dangerous for warmers that can cause short circuits.

*P: Yes, in SNCU the problem here is that there is no UPS [Uninterrupted Power Supply] on line at any SNCU. There is a lot of load. There are so many machines: about 40 warmers, three to four ventilators, 10–15 syringe pumps, phototherapy units, CPAP. There is so much load and they use the direct line for every instrument. Because of this there are a lot of problems with electricity. There is tripping of line, warmer gets burnt. But we keep maintenance regularly and because of us they do not face many problems … With electricity we have maximum problems. In one room there are 50 instruments. The line is not able to take on that much load. The RC board gets commonly damaged because of the power issue. That’s it. Nothing else. If the equipment is branded then there are no problems but if the equipment is local then there are problems. – AMC contractor*
*P: Yes, we have several issues in [Name of Hospital]. The high voltage, the machine goes off. I work with local fluctuations at [Name of Hospital]. We have to tell them, buy that, what is it called? I: Stabilizer? P: Yes, and one more UP I: UPS? P: Yes. I: The uninterrupted power supply, right? P: Yes. We have given that also. We have written that they should buy the stabilizer. It has been one year but they have not taken any action. I: So there haven’t been any changes since one year? P: Yes, the whole year, we have in SNCU /ICU these are crucial machines. And we have to install UPS. But till now they have not installed any power stabilizer. – AMC contractor*
*P: Yes, we will just divide the power issue into two subject groups. One group, the issue faced by all the equipment in the metro city. There, the power fluctuation is the minimum. And the other group would be the issue faced in the areas or the regions of rural areas where the power fluctuation is, you know, there is a huge power fluctuation. Since I am in India and India is not that developed country, if you go to see the rural areas you are going to face it and it is going to remain for next 10–20 years as well. So, for that we always suggest they use a good voltage stabilizer. So, what happens is that if it is a minor size, a minor voltage fluctuation, a good quality stabilizer will take care of it and the equipment is safe. Now if there is a lot of fluctuation, then that particular site might even enter your equipment to the 207 power supply card. So, what we have done in recent years is we have tried to use a very good quality 208 SMTS in the power supply. So that particular SMTS has reduced my issues with the power supply card. But then even an SMTS might have its own resistance and its own system which we do not know. So basically, we use EMI sockets, we use the SMPS so that this particular voltage fluctuation will not affect your equipment beyond the power supply card. - Equipment manufacturer*

A power supply card is an electric board that provides wiring for sensor inputs and power outputs. These electric boards or boxes come at a price and size depending on the type of electrical load expected. The 207 power supply card the participant mentions is a system used mainly for industrial applications and under certain non-explosive environmental conditions. Given the limitations of the power supply card, the participant is suggesting that they have used a Surface Mount Technology System (SMTS) that allows a circuit board to have customized circuits. The advantage of the SMTS is that because the circuit board is customized the manufacturer can adjust the power threshold of the circuit depending on the type of electrical work. However, the participant suggests that even the SMTS posed its own limitations which made them switch to a combination system of Electromagnetic Interference (EMI) sockets and Switch Mode Power Supply (SMPS). EMI sockets are small circuits that contain these voltage fluctuations. SMPS is a type of electric circuit and uses power from devices that are frequently turned on and off at a high frequency to maintain a desired level of voltage required by a piece of equipment. The participant is saying that a combination of EMI sockets and SMPS has worked most effectively in addressing equipment voltage.

*P: Each and every equipment has some backup, it would be like UPS backup. It shouldn’t shut down immediately. Every expensive machine should have a backup. If it shuts down immediately, it will cause damage to the small parts. Whatever machines, costlier machines, have some sort of back up at least for the initial part of the shutdown. If the lights are out, and the equipment is shut down immediately, it will decrease the life of the equipment. It will not function properly. It will cause some sort of problem after some time, say after one or two months, three months, six months, eight months that is going to cause problems and these machines will have less efficiency, more chances of damage. It will require calibration, and the number of visits will increase. – Pediatrician*

Participants indicated that the load on the circuit due to the warmers often trips the circuit. This is especially true during the winter months when all warmers are in use.

*The power points need to be good. If we use too many warmers, sometimes we turn on all the warmers at one time, then the MCPs [Multi-Chip Packages] trip and we have to turn them on again [She points to the MCP circuit. It is at a height so someone has to climb on a chair and turn them on again]. So, the [equipment] power plugs need to be good. We have a problem with that. We have to climb and do it. Electrician is not there for 24 hours. They take time to come so we climb up ourselves and do it. Stabilizers are there, so all equipment doesn't stop at one point. We have auto generators so we don’t have a problem. Just the power plugs. – In-charge nurse*

The participant indicates that when too many plugs of the warmer are plugged into the electrical sockets, it trips the *Multi-Chip Packages* because of the electrical load. Equipment manufacturers are concerned about the load on the system when several pieces of equipment are in use. The residential circuit board (RC board) is the main circuit board that gets damaged the most.

*Currently my suggestion is that according to the load of instruments in SNCU, the UPS should be used online. For any department in the hospital. And secondly, the users do not listen to us. They should follow our instructions while using the equipment. They do not handle the equipment properly. – Equipment manufacturer*
*Then you have that warmer. They do not close the machine from the main switch. There are a lot of fluctuations and the machine power supply goes off. – Equipment manufacturer*

The above participant indicates that when the warmer is not in use, it is not switched off from the electrical source (outlet switch). Rather the on/off switch on the equipment itself is used instead of the button where the equipment is plugged in. This implies that the power is not completely off, and therefore the equipment is susceptible to potential voltage fluctuations.

*Other participants mentioned the load is too much for the age of the electrical lines within the hospital.*

*There are a lot of problems. The electric line is 25–30 years old. So, then people are constantly charging, putting an adapter so how much can it take? You know how old electric lines are. All the lines are problematic. – In-charge nurse*
*I: And what about power fluctuations?*
*P: Look this is a very old institute. Sometimes, the wiring and all these things are very old. If we find any fluctuation, then we get it repaired. There is one separate electricity department who is functioning for [Names the Place] so we just call the person and he will do the work. – Pediatrician*

***Poor quality equipment and constant use lead to frequent breakdowns and need for repair.*** Several participants in the clinic setting, especially in government facilities, said that the quality of equipment is poor. As a result, the equipment frequently breaks down, leaving staff without the equipment necessary to provide optimal care to babies. While a facility may have 10 warmers on the books, five may not be working. Health care providers attribute the purchase of poor-quality equipment to bureaucratic decision-making based on low resources rather than on the quality of the equipment. A few providers indicated that some equipment is purchased that providers do not use or need.

*Our equipment [is] so overused that it gets spoilt … They are continuously on, with no break... Initially, the material we used to get was good. Nothing happened to it for at least five to six years. Now the material gets spoiled within five to six months. – Pediatrician*
*Sometimes even if they repair it, it will get spoiled. We have to call them again. It’s a waste of time. Their time and ours as well. They can’t do anything because the quality of the equipment is not that good. They say that these equipment are outdated and of poor quality and can be repaired to a certain extent, not beyond that. It has come from the government. – In-charge nurse*

Some participants said that poor quality warmers with faulty sensors can burn babies.

*I: And what do you feel that the warmer in this SNCU is performing that function optimally?*
*P: Yes … on the record they are performing optimally even the reports sent to the director’s office mentioned that they are performing optimally but in reality/ practically looks nothing like that … when the warmers are used in continuation for prolonged duration, [babies] get burned. – Pediatrician*
*P: Some cradles have a problem if the heater is overheated so that is a tension that the baby shouldn’t overheat and burn. The older ones are better. The ones kept here (points out) overheat a lot. I: Are they new? P: Yes. – Medical officer*

Staff identified warmers and other equipment as the most troublesome because of frequent malfunctioning.

*I: Which is the most troublesome equipment? P: Maybe warmer I: Why P: The temperature regulation can go wrong; the alarm may not ring sometime. – Medical officer*
*P: Warmer they repair instantly once we call. Our warmers are continuously in use. They come and repair it for us. Our skin probes can get problematic sometimes. They may not heat, or may overheat. – In-charge nurse*

## Facilitators

### Major themes: Power quality and equipment-related facilitators to providing optimal care for babies in the SNCU

The following major themes were identified as power quality and equipment-related facilitators to optimal care for babies in the SNCU:

- The power supply is stable due to generators.
- Equipment is user-friendly.
- The central government is investing in the power supply at the state and local levels, including the support to power audits.

***The power supply is stable due to generators.*** Clinical staff at one trust hospital were not concerned about the electrical supply because of the use of generators and stabilizers at this facility. All health facilities included in the study had working generators. However, these staff do not have a background in electrical engineering and many facilities do not have biomedical engineers on staff.

*I: Do you have problems with equipment function like electrical fluctuation? P: No because we have generators, we have what it’s called. I: generator and back up? P: Back up we have. What is used for fluctuation? I: Stabilizer. P: Yes, stabilizer and we have an inverter. I: So, you are well backed up? P: Yes. – Pediatrician*
*I: And what about electrical problems you encounter? P: Electrical problems are not there. There is no short circuiting at all. There is no problem with anything going wrong there ... – Pediatrician*
*I: Do you have electricity problems? P: No, we have generator backup. Even if it goes, it comes on within a minute. – In-charge nurse*

***Equipment is user-friendly.*** Providers stated that most of the equipment is easy to use, which enables staff to provide optimal care to babies. Although providers are not all trained to use the equipment when they are hired, most eventually are trained on the job.

*I: Which is easy equipment to use? Equipment or accessory such as tube or syringe… P: In equipment, warmer and Phototherapy Unit are easy. I: Why are they easy for you? P: It’s not a complicated system. It can be managed with two buttons. – Medical officer*
*I: Sir, according to you what equipment or accessories are easy to use? P: Easy to use is the warmer, syringe pump because all our staff have been given proper training on how to handle the instruments. They know how to adjust the rates, how to start and how to stop the warmer, syringe pump, glucometer. – Pediatrician*
*I: Which is easy equipment to use? P: Phototherapy unit is the easiest unit, because it doesn’t need supervision; for example, in a warmer you have to pay attention or there are chances that the baby might burn. We will have to keep a sister for supervision. But we don’t have that. So, we can’t use it. Phototherapy is not like that. It doesn’t need supervision. – Medical Officer*

***The central government is investing in the power supply at the state and local levels, including the support to power audits.*** Government officials indicated that the Government of India has supported electrification of the country to ensure key health services are not interrupted, including the cold chain for vaccines. The government has also invested in generators and solar-powered refrigerators to ensure power availability.

*I will not put it as an important area because the Government of India is very much concerned about electrification. Because this electricity is required not only for equipment in the SNCU or NBSU* [Newborn Stabilization Unit] *, but they are also required for other things like cold chain equipment across the country. Vaccination is one of the highest priority programs in our country. And we have been able to provide electricity to the remotest part of the country. Because the immunization program pushed us to make sure that electricity is available whether in the form of solar refrigerators or other things or alternative means, like you have these what we call, generators have been provided. So, I think we don’t put it as a major challenge. This is being addressed as a cost cutting issue. And when the generators are available for providing electricity to the refrigerators or the cold chain equipment, I will say that generators can be utilized for the SNCU equipment. I don’t find this to be a major challenge. Maybe I don’t know, in your interviews, there might be exceptional situations. But, generally, I will not put it as one of the priority areas. – Ministry of Health representative*
*That is a great thing … this power audit is the most important thing for the unit. It happens at the state as well as the local authorities for any kind of breakdown of the system. – Ministry of Health representative*
*We are doing power audits in these SNCUs about the power supply and we are also even giving a separate generator facility to these units. Means they have dedicated power supply, apart from the regular unit. If the SNCU unit is established in the district hospital, then there will be two power generators: one for the unit and one for the district hospital. So, we are giving facility wise generators also. – Ministry of Health representative*

## Participant recommendations

### Major themes: Participant power quality and equipment-related recommendations to improve care for babies in the SNCU

Participants made several concrete recommendations to improve care for babies in the SNCU:

- Procure stabilizers to ensure equipment does not get damaged.
- Improve quality/durability of equipment to withstand constant use.
- Conduct regular ‘proactive maintenance’ for SNCU equipment.
- Organize local power audits to discern and address the causes of power fluctuations.

***Procure stabilizers to ensure equipment does not get damaged.*** AMC contractors recommended that the hospitals procure UPS/stabilizers to prevent surges from damaging the equipment. A Ministry representative recommended a state power audit.

*P: Currently my suggestion is that according to the load of instruments in SNCU, the UPS should be used online for any department in the hospital. And secondly, the users do not listen to us. – AMC Contractor*

***Improve quality of equipment to withstand constant use.*** Poor quality equipment leads to frequent breakdowns and under functioning, leaving a shortage of equipment in the SNCU and potential for infection in babies due to equipment sharing. Several participants recommended investing in high quality equipment durable enough to withstand constant use. This investment requires political will and appropriate budgeting by hospital administrators who themselves must request funding from higher authorities. Some participants recommended upgrading to newer equipment with better lighting and alarm systems.

*I: How do you think things can be improved at the equipment level in terms of manual, design, user friendliness? P: It will be good to have advanced equipment, some of our phototherapy units and warmers are recent so it’s good. But some are old so we can’t rely on them much. We have LED phototherapy; it is more effective. Compared to the old tube light ones they are less efficient. According to recent advances, we should get equipment accordingly. – Medical officer*
*I: Is there anything you would like to change in terms of equipment user friendliness or design? How can the equipment be made more efficient? P: We just want the equipment to work properly, no matter what. – In-charge nurse*

***Conduct ‘proactive maintenance’ of SNCU equipment.*** AMC contractors should routinely check the equipment for any damage and repair as needed. Detecting a problem early would reduce the number of broken equipment.

*I: Do you have any recommendations to improve the SNCU efficiency? P: Regarding equipment? I: Yes. P: It's the same that whatever we are installing in the SNCU, we should get proper response from the company and that person should come here and regularly check the instruments; whether they are working to detect problems in the early phase. We come to know when a problem is major. Voltage and current and all those problems can be identified. I: So, you are saying they should come without calling? P: Yes, so they can detect early. We call them when we get a major problem and it takes time to resolve. It’s like proactive maintenance. They should keep maintenance of the machines. – Medical officer*

***Organize power audits at the state and local levels.*** Since health-related issues are the responsibility of the state, not the national government, representatives of the Ministry of Health react to the requests from the state. One MOH representative suggested that each state request power audits.

*P: As I have mentioned earlier also the priority of newborn survival is one of utmost priority of the ministry of health and health in India is a state subject. So, we are just here at the ministry level giving approval based on certain proposals of the state. If the state wants the power audit or some kind of structure of newborn services in the state, we have no objection. We have given approval from here first thing. Second thing is as you have mentioned if there will be power audit system that generates some kind of data regarding the quality of the NIC [non-interrupted connection] to the unit, how many hours the power shut down, or any other thing, it will be helpful for the state as well as the national level – Ministry of Health representative*

## Discussion

Neonatal equipment to monitor and treat certain conditions is critical to the survival of babies in neonatal units worldwide. Our study found several barriers to the function and effective use of medical equipment in neonatal care units, the most important of which were poor power quality straining old and poor-quality equipment and inefficient and/or poor-quality equipment repair that resulted in limited unavailability of critical equipment. Facilitators of neonatal equipment use included reliable and steady power, user-friendly equipment, and the ability of the state to conduct power audits at the state and local levels. Recommendations focused on what could be done to improve the quality of the equipment by manufacturers, the quality and timeliness of maintenance and repair by AMC contractors, and the quality and stability of the power supply by the facilities and the government.

There is a paucity of data on the effects of poor quality and unstable power on the proper functioning of neonatal care equipment in India. To our knowledge, this is the first study to document the understanding among stakeholders of these effects on crucial neonatal equipment. Much can be done in the short term, including provisioning each unit with its own generator, uninterrupted power supply, and power conditioners, regular training on equipment use, proactive repairs, and power audits. However, overall improvements in the quality of the power at local and district levels will take time. Manufacturers play an important role in fully comprehending the real-world challenging circumstances in which their equipment will be used. Manufacturers must build equipment that is safe and able to tolerate constant use, sags, surges and spikes in the power supply.

While hospital staff did not perceive the quality of the electrical supply to be a problem, AMC contractors, bioengineers, and equipment manufacturers indicated that the frequent sags, surges and spikes may be compromising the integrity of the equipment. Equipment manufacturers and AMC contractors recommended uninterrupted power supply (UPS) and stabilizers be installed in all facilities to protect equipment. Surges, spikes, swells, sags or brownouts, noise and outages, all common in low- and middle-income countries, can damage sensitive medical equipment and put patients at risk ( https://www.gradianhealth.org/). Facilities need to invest in protective devices such as UPS, stabilizers, and power conditioners to mitigate and prevent damage to neonatal care equipment. State power audits would help to determine how best to address the power quality problem.

Other studies have reported poor quality equipment, frequent breakdowns, and slow repairs of equipment in the neonatal units in India ^4^. Consequences of fewer functioning pieces of equipment include sharing of equipment between infants that increases the risk of nosocomial infection. AMC contractors must understand how unstable power can damage each piece of equipment and proactively monitor the equipment to ensure small shocks are addressed before the equipment fails completely. AMC contractors also need to be held accountable for regular maintenance and timely repairs, and government facilities need to invest in procuring safe and reliable equipment.

Equipment end-users in the clinic must be properly trained to use the equipment and to detect under-functioning and stress on the equipment. Our study agreed with other studies that have reported that lack of training is a key barrier to proper use of equipment and optimal care for neonates ^4, 13– 15^. Training staff on the correct and optimal use of neonatal equipment prior to caring for sick neonates needs to be prioritized, as does a new focus on retraining to ensure staff are up to date on the latest equipment.

Other studies in India have documented the improvements in the quality and reliability of neonatal equipment over time ^3^ as well as the expansion of access to care at SNCUs throughout India ^4, 9^. However, compared to government facilities, trust and private hospitals appear to have better access to higher quality equipment and conduct more regular training on the proper use of the equipment.

This study has a number of strengths. First, our interviewers were highly trained and have several years of experience conducting qualitative research in the Indian context. Our analytical team systematically coded and analyzed the data using an intensive Grounded Theory approach to identify the major themes, patterns, and relationships between themes that focused on power quality and SNCU equipment. This study also has a few limitations. First, the results of this study cannot be generalized to other types of neonatal facilities (e.g., neonatal intensive care units (NICUs) or to facilities beyond our study catchment area in a district of Maharashtra State. The majority of our participants were clinical staff and their knowledge of power quality was limited. We interviewed two biomedical engineers (working for equipment manufacturers) regarding power quality and efforts to maintain that quality in the face of sags, surges, and spikes, in part due to the impact of the COVID-19 pandemic that limited time available during the grant. In addition, since SNCU directors selected eligible staff to participate in interviews and focus group discussions, it is possible that the individuals deemed eligible might not represent the opinions of other staff who were not selected.

## Conclusion

Our study puts a new focus on the consequences of poor power quality, not just power availability as a cause of compromised equipment, e.g., micro- and macro-level damages to the equipment that are not detected by the end users. Generators are increasingly available and may ensure the power goes back on, but most SNCUs do not have uninterrupted power supply /power conditioners and stabilizers to prevent shocks to the equipment from sags, surges and spikes. Further research is needed to understand a cost-effective way to improve power quality at the central and/or facility level and whether this translates to reductions in neonatal mortality in at-risk babies in SNCUs. For more information, please see website: https://sites.bu.edu/BB4B

## Data availability

### Underlying data

Zenodo: Underlying data for ‘Poor power quality is a major barrier to providing optimal care in special neonatal care units (SNCU) in Central India’, https://doi.org/10.5281/zenodo.5737387 ^11^.

This project contains the following underlying data:

- Quotes matrix_BB4B.xlsx

The audio transcripts are not openly available for data protection reasons because, despite removing identifiable information such as names and organizational affiliations, we risk revealing individual identifies through the interview responses. As part of the written consent agreement with participants, we assured them of anonymity when presenting synthesized findings. Requests for data must be provided in writing and should include a detailed rationale. All requests must be made by email to Lisa Messersmith at ljmesser@bu.edu. Access may be granted for legitimate research purposes. The Boston University Medical Center IRB will review all requests.

### Extended data

Zenodo: Extended data for ‘Poor power quality is a major barrier to providing optimal care in special neonatal care units (SNCU) in Central India’, https://doi.org/10.5281/zenodo.5736929 ^12^.

This project contains the following extended data:

- FGD consent form nurses.pdf
- FGD consent form residents.pdf
- FGD guide staff nurses and padiatricians.pdf
- KII consent form annual maintenance contractors.pdf
- KII consent form biomedical engineers.pdf
- KII consent form doctors.pdf
- KII consent form manufacturers.pdf
- KII consent form Ministry of Health.pdf
- KII consent form nurses.pdf
- KII guide administrators.pdf
- KII guide annual maintenance contractor.pdf
- KII guide biomedical engineers.pdf
- KII guide health providers.pdf
- KII Ministry of Health.pdf

Data are available under the terms of the Creative Commons Attribution 4.0 International license (CC-BY 4.0)

## Consent

Written informed consent for publication of the participants’ details was obtained from the participants.
