# Supplementary material for: Poor power quality is a major barrier to providing optimal care in special neonatal care units (SNCU) in Central India
Source: Gates Open Res. 2022 Apr 27;6:58. [Version 1] doi: 10.12688/gatesopenres.13479.1 (PMC10293610; doi:10.12688/gatesopenres.13479.1)
Supplement: Peer review history

# Peer review: Reviewer response for version 1

**DOI:** 10.21956/gatesopenres.14739.r33497

**Published:** 2023-06-26

**Authors:** Julia Johnson

**Affiliations:** 1 Division of Neonatology, Department of Pediatrics, Johns Hopkins University, Baltimore, MD, USA

The authors present their work on identification of barriers to care delivery, with a focus on medical equipment, in special neonatal care units in central India, which involved focus group discussions and key informant interviews with frontline clinical staff and Ministry of Health personnel, as well as contractors and manufacturers of such devices and equipment. The methods section clearly describes the study’s approach, using Grounded Theory. Major themes identified included equipment-related barriers, including the impact of an unstable power supply and poor equipment quality/needs for repair. Focus group and key informant interview participants provided recommendations to improve care, to include procurement of stabilizers, improvement of equipment quality, conduct of regular equipment maintenance, and use of local power audits.

 The inclusion of contractors and manufacturers in this qualitative study addresses a gap in the literature, as the focus of neonatal care delivery is often on the clinical aspects of care, rather than the infrastructure required to ensure care delivery. The authors recognize a disconnect between barriers identified by this important group of stakeholders and those identified by healthcare workers, as unit-based staff were less likely to recognize or identify the impact of electrical supply quality on care delivery.

 A strength of this study is the inclusion of concrete action items to address some of the identified barriers. However, the capacity to implement these changes is of course resource-dependent and will partially depend on support by the government and other entities outside of the healthcare facility, in particular for improvement of electrical supply quality. Innovative approaches to reducing dependence on local power supply and improving quality of equipment for healthcare delivery will be needed while larger infrastructure improvements are addressed.

 There are of course other barriers related to the use and functionality of equipment in neonatal care delivery and the authors could consider a summary of these, if they were identified during the focus groups or key informant interviews. Additionally, how these barriers may interact with the larger work system and other aspects of neonatal care delivery could be presented visually to aid in contextualizing this work.

Is the work clearly and accurately presented and does it cite the current literature?

Yes

If applicable, is the statistical analysis and its interpretation appropriate?

Yes

Are all the source data underlying the results available to ensure full reproducibility?

Partly

Is the study design appropriate and is the work technically sound?

Yes

Are the conclusions drawn adequately supported by the results?

Yes

Are sufficient details of methods and analysis provided to allow replication by others?

Yes

Reviewer Expertise:

Neonatology, global health, infection prevention and control, quality improvement

I confirm that I have read this submission and believe that I have an appropriate level of expertise to confirm that it is of an acceptable scientific standard.

# Peer review: Reviewer response for version 1

**DOI:** 10.21956/gatesopenres.14739.r33501

**Published:** 2023-06-20

**Authors:** Terence Leung

**Affiliations:** 1 Medical Physics and Biomedical Engineering, University College London, London, UK

This paper describes a qualitative research study on issues affecting the special neonatal care units in Central India. The researchers conducted Focus Group Discussions and Key Informant Interviews with stakeholders including care providers, hospital and government administrators, equipment manufacturers and maintenance contractors. The interviews were transcribed and the data were then uploaded into NVivo for coding. The research methodology was based on Grounded Theory. The investigation concluded poor power quality as the main barrier to providing optimal care in special neonatal care units.

 The paper was very well written, with clear explanation and discussion on the background, methodology, data analysis and results. Details into what poor power quality actually meant were described providing important insights into the issue. I just have a few queries as listed below.

 During the theoretical sampling, did you see an alternative core category emerging (other than the poor power quality theme) in the codes? I asked this because there could be other barriers, e.g., bureaucracy in managing equipment, lack of financial resources, etc. so I wonder if poor power quality was the single major barrier, or there were other equally significant but slightly less pressing barriers?

 The iterative nature of Grounded Theory means that new knowledge is added continuously as the theory is being developed. Did you revise or add participants (e.g. equipment manufacturer) during the study to strengthen your theme? I asked this because it seemed from the paper that you planned all the interviews in advance so I wonder if you had taken the advantage of the flexibility offered by Grounded Theory to steer the mix of your participants during theoretical sampling.

 Finally, I wanted to highlight two terms that may lead to misunderstanding for readers from other parts of the world. The term MCPs or Multi-Chip Packages is normally used to describe a packaging technique adopted in microelectronics assembly. Perhaps you can clarify whether MCP (mentioned on page 6) in India is similar to MCB (miniature circuit breaker), a standard electrical protection device that trips when excessive current is detected? Also, is the RC board (residential circuit board) in India similar to RCB (residual current breaker) that trips when current leakage is detected in the earth wire?

Is the work clearly and accurately presented and does it cite the current literature?

Yes

If applicable, is the statistical analysis and its interpretation appropriate?

Not applicable

Are all the source data underlying the results available to ensure full reproducibility?

Partly

Is the study design appropriate and is the work technically sound?

Yes

Are the conclusions drawn adequately supported by the results?

Yes

Are sufficient details of methods and analysis provided to allow replication by others?

Yes

Reviewer Expertise:

Physiological monitoring and medical device development for neonates.

I confirm that I have read this submission and believe that I have an appropriate level of expertise to confirm that it is of an acceptable scientific standard.
